# Clinical and Biochemical Correlates of Serum L-Ergothioneine Concentrations in Community-Dwelling Middle-Aged and Older Adults

**DOI:** 10.1371/journal.pone.0084918

**Published:** 2014-01-02

**Authors:** Salvatore Sotgia, Angelo Zinellu, Arduino A. Mangoni, Gianfranco Pintus, John Attia, Ciriaco Carru, Mark McEvoy

**Affiliations:** 1 Department of Biomedical Sciences, University of Sassari, Sassari, Italy; 2 Department of Clinical Pharmacology, School of Medicine, Flinders University, Adelaide, Australia; 3 Level 3 Hunter Medical Research Institute, School of Medicine & Public Health, University of Newcastle, Newcastle, NSW, Australia; 4 Centre for Clinical Epidemiology and Biostatistics, University of Newcastle, Newcastle, NSW, Australia; University of Catania, Italy

## Abstract

**Background:**

Despite the increasing interest towards the biological role of L-ergothioneine, little is known about the serum concentrations of this unusual aminothiol in older adults. We addressed this issue in a representative sample of community-dwelling middle-aged and older adults.

**Methods:**

Body mass index, estimated glomerular filtration rate, serum concentrations of L-ergothioneine, taurine, homocysteine, cysteine, glutathione, cysteinylglycine, and glutamylcysteine were evaluated in 439 subjects (age 55–85 years) randomly selected from the Hunter Community Study.

**Results:**

Median L-ergothioneine concentration in the entire cohort was 1.01 IQR 0.78–1.33 µmol/L. Concentrations were not affected by gender (P = 0.41) or by presence of chronic medical conditions (P = 0.15). By considering only healthy subjects, we defined a reference interval for L-ergothioneine serum concentrations from 0.36 (90% CI 0.31–0.44) to 3.08 (90% CI 2.45–3.76) µmol/L. Using stepwise multiple linear regression analysis L-ergothioneine was negatively correlated with age (rpartial = −0.15; P = 0.0018) and with glutamylcysteine concentrations (rpartial = −0.13; P = 0.0063).

**Conclusions:**

A thorough analysis of serum L-ergothioneine concentrations was performed in a large group of community-dwelling middle-aged and older adults. Reference intervals were established. Age and glutamylcysteine were independently negatively associated with L-ergothioneine serum concentration.

## Introduction

It is generally accepted that damage caused by free radicals is a hallmark of ageing with potential clinical consequences [1]. In humans, a complex of enzymatic and non-enzymatic antioxidants reacts to neutralize these damaging molecules and exogenous substances may play a crucial role in this context [1]. Although its specific physiological role and the consequences of its deficiency are unclear, among exogenous compounds with possible antioxidant activity, the interest toward ergothioneine (ERT; 2-mercaptohistidine trimethylbetaine) has been increasing in the last years. ERT is an unusual hydrophilic low-molecular-weight thiol [2], exclusively synthesized in few organisms such as non-yeast-like fungi including edible mushrooms [3], mycobacteria [4] and cyanobacteria [5].

Early studies showed that humans and other vertebrates are unable to biosynthesize ERT, and that in mammals it is acquired solely by dietary means [2]. A specific organic cation transporter protein (ETT), codified by the SLC22A4 gene [6], is responsible for its accumulation in the body at millimolar levels [7], [8]. ETT is strongly expressed in fetal liver, small intestine, trachea, kidney, cerebellum, lung, and cells of the myeloic lineage such as erythrocyte progenitor cells in bone marrow and monocytes [8]. Cells lacking ETT do not accumulate ERT and its uptake does not appear to be related to dietary sources but, rather, to the expression of ETT mRNA [2]. The existence of a specific transporter suggests a biological role for ERT. Moreover, case-control studies have showed associations between polymorphisms in the SLC22A4 gene and susceptibility to some chronic inflammatory diseases such as Crohn’s disease [9], ulcerative colitis [10], rheumatoid arthritis [11], and Type I diabetes [12]. ERT concentrates especially in mitochondria and in cells and tissues normally exposed to oxidative stress and those involved in an inflammatory response [3]. These observations suggest that ERT might have antioxidant and scavenging activities, similarly to the main water-soluble antioxidant thiol glutathione [13]. ERT chemistry, however, differs from conventional sulfur-containing antioxidants. ERT, in fact, exists as a tautomer between its thiol and thione forms, with the latter predominating under physiological conditions [7]. Consequently, ERT shows a peculiar stability and reactivity compared to other naturally occurring thiols, since it does not autooxidize [6], does not form disulphides and mixed disulphides [14], requires a more severe oxidative stress to oxidize and does not promote the classical Fenton reaction [6].

The knowledge regarding tissue ERT concentration distribution at population level is limited to one study in 400 healthy male individuals [15]. Reported erythrocyte ERT concentration, averages and ranges, differed across age groups, i.e. 65.42–109.03 µM (1–10 years), 161.36 µM (11–18 years), 100.30–130.83 µM (19–50 years) and 122.11 µM (≥51 years) [15]. No data, instead, are available on ERT concentrations in human serum. This is possibly due to the lack of reliable analytical methods with adequate sensitivity as serum ERT concentration is much lower vs. erythrocytes. To this regard, we have recently developed the first capillary electrophoresis-laser-induced fluorescence (CE-LIF) method for the rapid evaluation of plasma or serum ERT concentrations [16]. The assay method was able to quantitatively detect and measure ERT concentrations as low as 0.27 µmol/L, with a limit of detection of 90 nmol/L.

Given the availability of this new sensitive assay method, we studied the distribution of serum ERT concentrations in an established epidemiological cohort of human ageing, the Hunter Community Study (HCS) [17], and sought to identify clinical and biochemical correlates.

## Materials and Methods

### Study Population

Participants were a representative sample of the Hunter Community Study (HCS), a population-based cohort study to assess the impact of clinical, genetic, biochemical, socioeconomic, and behavioural factors on human ageing [17]. The cohort consisted of community-dwelling men and women aged 55 to 85 years residing in Newcastle (New South Wales, Australia), randomly selected from the electoral roll and contacted between December 2004 and December 2007. Of the 9,784 individuals who received the invitation letter, a total of 3,253 actually took part (response rate 44.5% after removing incorrect addresses and non-contacts) and completed a series of self-reported questionnaires, attended a clinic visit, consented to linkage of health records and to undergo to a series of clinical and biochemical measures. The sample for this investigation (n = 500) was derived from the initial cohort by simple random sampling. Of the 500 subjects randomly selected, complete exposure and outcome data at baseline were available for 439 subjects, 227 males and 212 females. The comparison of this sample with the entire cohort showed no significant difference for a range of clinical, biochemical, socioeconomic, and behavioural factors (data not shown), ensuring that the sampling was representative of the original cohort. Approval to conduct the research was granted by the University of Newcastle Human Research Ethics Committees, it was performed in accordance with the guidelines of the Declaration of Helsinki and, after being informed, all participants gave written informed consent.

### Blood Sample Collection and ERT Analysis

Blood was collected into tubes without anticoagulant and centrifuged at 4°C and 3,000×g for 10 min to separate serum, which was stored for three years at −80°C before analysis. There was no previous freeze-thaw cycles prior to analysis. Serum ERT concentration was measured by capillary electrophoresis-laser-induced fluorescence method as recently described by Sotgia et al. [16]. Briefly, to precipitate the proteins, a 100 µL-volume of acetonitrile was added to 100 µL of sample and mixed thoroughly by vigorous vortex-mixing. After centrifugation at 17,000×g for 10 min at room temperature, 50 µL of the derivatization reagent were added to 150 µL of supernatant and, after vigorous vortex-mixing, reaction mixture was left in a light-protected area for 30 min at room temperature. Finally, samples were diluted with water fifty times and analysed by CE-LIF. The limits of detection and of quantification were 0.09 and 0.27 µmol/l, respectively. The variations for intra- and inter-assay precision were around 6 RSD%, and the mean recovery accuracy close to 100% (96.11%).

### Demographic, Clinical and Biochemical Variables

This study examined the association between serum ERT with age, gender, body mass index (BMI), estimated glomerular filtration rate (eGFR), and the thiols homocysteine (Hcy), cysteine (Cys), glutathione (GSH), cysteinylglycine (CysGly), glutamylcysteine (GluCys), and taurine (Tau). Body mass index was calculated using height and weight measurements obtained at baseline and eGFR was calculated using the Modification of Diet in Renal Disease formula [18]. Serum concentrations of the thiols homocysteine (Hcy), cysteine (Cys), glutathione (GSH), cysteinylglycine (CysGly), glutamylcysteine (GluCys), and taurine (Tau) were measured previously by CE-LIF methods, as described by Zinellu et al. [19], [20]. eGFR has been shown to be an independent determinant of plasma concentrations of Hcy, Cys and CysGly. Therefore, it was used to assess potential associations between ERT concentrations and kidney function [21], [22]. In view of the dietary origin of ERT, BMI was used as a surrogate marker of nutritional status. BMI and other measures of adiposity, in fact, have been associated to concentrations of biochemical markers of nutritional status. [23] A self-administered questionnaire was used to collect information of existing chronic medical conditions such as cardiovascular disease, type 2 diabetes, arthritis and depression. Study participants were asked if they had ever received a physician diagnosis of a range of medical conditions and the age that they received the diagnosis.

### Statistical

Statistical analyses were performed using MedCalc for Windows, version 12.5 64 bit (MedCalc Software, Ostend, Belgium) and SPSS for Windows, version 14.0 32 bit (IBM Corporation; Armonk, NY, USA). Data were tested for normality of distribution using the Kolmogorov-Smirnov test. Data were presented as either mean±SD or median and interquartile range (IQR) as appropriate. Non-normally distributed variables were log10-transformed prior to being used with parametric tests, and the normal distribution of the residuals was checked to assess the goodness of fit of the transformations. Differences between groups were compared by non-parametric Mann-Whitney U Test or by independent t-test as appropriate. Spearman rank correlation coefficients were used to characterise associations between serum ERT concentration and continuous clinical, biochemical and demographic variables. Partial correlation and stepwise multiple linear regression analysis was used to assess the contribution of different variables to serum ERT concentration. Multicollinearity was tested by measuring the tolerance and the variance inflation factor values for each analysis. A 2-sided P value of 0.05 indicated statistical significance.

## Results

**Figure 1:**
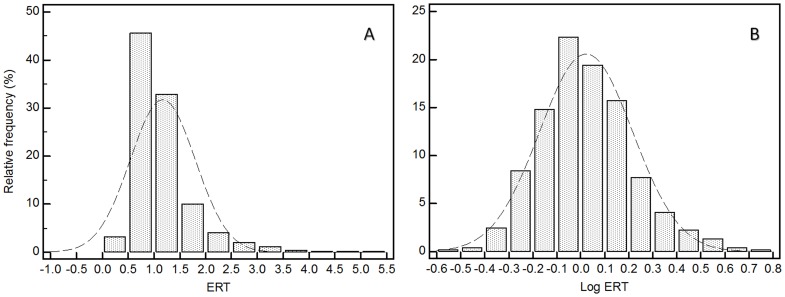
Distribution of ERT concentrations in the population (A) before and (B) after log10 transformation. Lowest and highest values were, respectively, 0.26 and 5.06 µM. Geometric mean after back transformation of log-transformed data was 1.05 µM and 95% CI for the mean was 1.01 to 1.10 µM. Median was 1.01 µM and 95% CI for the median 0.96 to 1.06 µM.

**Figure 2:**
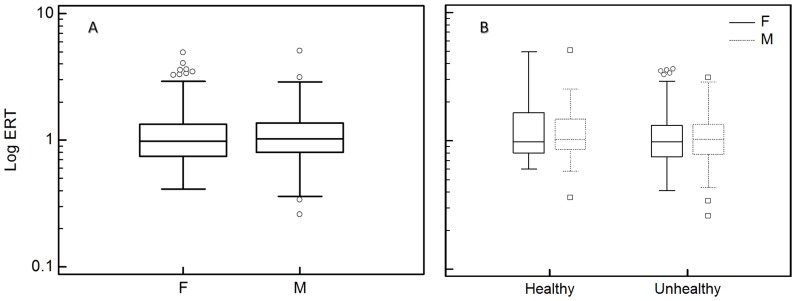
Distribution of ERT concentrations in the HCS sample after (A) grouping according to the gender and (B) to the presence or absence of disease and by gender. The lower and upper lines of the box correspond to the 25th and 75th percentiles, respectively. The line in the middle of the box represents the median. The error bars are situated at <1.5 times the interquartile range (ie, 75th percentile to 25th percentile) from the upper and lower lines of the box. □, ○: outliers.

Demographic, clinical, and biochemical characteristics of the study sample are outlined in Table 1. ERT serum concentration distribution was skewed, with ∼45% of the study population having concentrations between 0.5 and 1.0 µmol/L (Figure 1). Median ERT serum concentration was 1.01 IQR 0.78–1.33 µmol/L. No significant gender-related differences were observed (females 0.97 IQR 0.75–1.32 µmol/L vs. males 1.04 IQR 0.80–1.36 µmol/L, P = 0.41) (Figure 2A). The majority of participants (81.8%) had at least one chronic condition, such as hypertension (59.9%), angina (5.8%), osteoarthritis (28.7%), rheumatoid arthritis (6.7%), diabetes (13.6%), thyroid problems (12.3%), depression (24.8%), emphysema (13.9%), and osteoporosis (7.0%). Moreover, a small proportion had suffered a myocardial infarction (7.5%) or a stroke (3.6%). Only 18.2% of the participants did not suffer from any apparent disease.

**Table 1:** Clinical, demographic, and biochemical characteristics of the 439 older subjects.

	All (n = 439)	Healthy (n = 80)	Unehalthy (n = 359)
Females *%*	48.3	42.5	49.6
Age *years, median (IQR)*	64 (60–70)	62 (59–66)	65 (60–71)
ERT *µM, median (IQR)*	1.01 (0.78–1.35)	0.99 (0.84–1.52)	1.01 (0.77–1.33)
BMI *Kg/m^2^, median (IQR)*	28.0 (25.8–31.3)	26.7 (25.4–29.6)	28.4 (25.9–31.8)
eGFR^a^ *ml/min, mean±SD*	78±16	80±14	78±17
Tau *µM, median (IQR)*	62.40 (52.25–83.67)	60.13 (52.47–79.15)	63.57 (52.20–84.20)
CysGly *µM, median (IQR)*	29.01 (25.50–33.18)	28.73 (25.81–32.08)	29.12 (25.43–33.48)
Hcy *µM, median (IQR)*	9.14 (7.94–11.06)	8.65 (7.58–10.10)	9.28 (8.02–11.26)
Cys *µM, mean ±SD*	188.99±37.10	172.95±27.38	192.55±38.05
GSH *µM, median (IQR)*	3.87 (2.93–5.28)	3.92 (3.02–5.82)	3.87 (2.91–5.21)
GluCys *µM, mean ±SD*	4.40±1.20	4.37±1.26	4.41±1.17

^a^ Calculated using the modification of diet in renal disease formula.

Those with at least one chronic medical condition were classified as unhealthy (N = 359) while those without were classified as healthy (N = 80). The presence of a chronic medical condition was not associated with a significant difference in ERT serum concentrations [healthy subjects (n = 80) 0.99 IQR 0.84–1.52 µmol/L vs. unhealthy subjects (n = 359) 1.01 IQR 0.77–1.33 µmol/L, P = 0.15]. In the subgroups obtained by a further split according to the gender, ERT serum concentration was slightly higher in males than females but the difference was not statistically significant both in healthy (P = 0.99) and unhealthy (P = 0.41) groups (Figure 2B). In view of the similar ERT serum concentrations in healthy vs. unhealthy groups and males vs. females, further analyses were performed by considering the cohort as a whole. As displayed in Table 2, Spearman rank correlation tests showed a statistically significant negative correlation between ERT serum concentration and age (rho = −0.165; P = 0.0005), Hcy (rho = −0.139 P = 0.0036), Cys (rho = −0.111 P = 0.02) and GluCys (rho = −0.202 P<0.0001). ERT concentrations were also negative correlated with GSH (rho = −0.0927 P = 0.05) and positively correlated with eGFR (rho = 0.149; P = 0.0018). No statistically significant correlations were observed with either BMI (rho = 0.0666 P = 0.16), CysGly (rho = −0.0724 P = 0.13) or Tau (rho = 0.0101; P = 0.83).

**Table 2:** Correlation analysis.

	ERT	P-value
Age	rho = −0.165	0.0005
CysGly	rho = −0.072	0.1314
Hcy	rho = −0.139	0.0036
Cys	rho = −0.111	0.0207
GSH	rho = −0.092	0.0539
GluCys	rho = −0.202	<0.0001
Tau	rho = 0.010	0.8337
eGFR	rho = 0.149	0.0018
BMI	rho = 0.067	0.1633

Spearman rank correlation between ERT concentrations, demographic factors, and biochemical variables.

To detect potentially confounding variables, we performed a partial correlation analysis by using gender and healthy state as covariates and age, BMI, eGFR, Tau, Hcy, GSH, Cys, CysGly, and GluCys as covariates or as variables. This analysis confirmed only the statistically significant negative association of ERT serum concentration with age (rpartial = −0.105; P = 0.03) and with GluCys (rpartial = −0.113; P = 0.02). When redundant, non-significant variables were removed by using stepwise multiple regression analysis (Forward+Backward method), independent and stronger associations with age (rpartial = −0.150; P = 0.0022) and with GluCys (rpartial = −0.132; P = 0.0060) were observed (Table 3). The goodness of the model also improved as the F-ratio and significance magnitude increased from 3 (P = 0.001) to 12 (P<0.001). By considering only the healthy group (n = 80), the reference interval calculated by the robust method (CLSI C28-A3) [24] for smaller sample sizes ranged from 0.36 (90% CI 0.31–0.44) to 3.08 (90% CI 2.45–3.76) µmol/L.

**Table 3:** Stepwise regression of serum ERT concentrations.

	B coefficient (95% CI)	rpartial	P-value
Log Age	−0.6 (−0.98 to −0.22)	−0.150	0.0022
GluCys	−0.02 (−0.04 to −0.01)	−0.132	0.006

The variables entered in the model were healthy status, gender, age, BMI, eGFR, CysGly, Hcy, Cys, GSH, GluCys, and Tau.

## Discussion

Low-molecular-weight thiols have been extensively investigated in older adults [25]. By contrast, little is known about ERT serum concentrations in this group. Studies have focused mainly on its content in foods, animals and in the whole blood or erythrocytes of healthy subjects or patients with autoimmune diseases. We demonstrated that ERT is measurable in serum and, differently from what is reported in the literature for the plasma ERT levels, the concentration in serum appears generally lower than that expected considering from 2 to 9 times the content of red blood cells [14]. Due to the lack of direct measurements of ERT concentrations in erythrocytes or whole blood we could not directly assess ratios between these compartments and serum. However, serum ERT concentration in the HCS sample was consistent with plasma ERT levels measured by our group on a small set of age-matched subjects [16], [26], where the whole blood/plasma ratio was an average 40. However, given the circumstantial nature of this result, a more depth investigation of the ratio is currently under investigation in our laboratory.

Despite the analogies suggested by some authors with GSH, e.g. the antioxidant activity and the similarities between intracellular and extracellular concentrations, no independent association was found in this study between ERT and GSH. Similarly, no independent association was observed with Tau, the other antioxidant considered in the study.

Although bivariate analysis showed associations between ERT serum concentration and other aminothiols (Hcy, Cys, and GluCys), stepwise regression only showed independent negative associations with GluCys. Age was the only other variable showing independent negative associations with ERT serum concentrations. Although ERT concentration reduces with age, and indeed the median age in the unhealthy group was higher vs. healthy subjects (66.0±7.7 years vs. 63.5±6.1 years), ERT concentrations are unlikely to serve as an index of health status. The ERT concentrations between two groups were, in fact, not significantly different, and almost all ERT values in unhealthy subjects (∼98%) lie within the reference interval (0.36–3.08 µmol/L). The decreased levels of ERT with age could reflect either a modification of dietary habits or alterations in the expression of the SLC22A4 gene.

The negative association between ERT and GluCys is not easy to explain, also in view of the lack of a clear relation with GSH. GluCys, the immediate precursor of GSH, is synthesised by the enzyme glutamylcysteine synthetase in the γ-glutamyl cycle. The activity of glutamylcysteine synthetase is limited by availability of Cys and glutamate, and is inhibited by GSH through a feedback mechanism [27]. Recent studies have showed that GluCys is a thiol-redox regulator that efficiently detoxifies mitochondrial ROS by acting as glutathione peroxidase-1 cofactor [28] and inhibits oxidative stress in human endothelial cells [29]. Moreover, GluCys modulates the trans-membrane transport of several cationic amino acids [30]. ERT might act similarly to GSH, i.e. by inhibiting the glutamylcysteine synthetase activity. This might explain the weak association between ERT and GSH. However, further experimental support is necessary to corroborate these findings and to provide mechanistic insights.
